# A Review of the Latest Guidelines for Diagnosing and Managing Asthma in Children in the United States and Canada

**DOI:** 10.7759/cureus.68135

**Published:** 2024-08-29

**Authors:** Okelue E Okobi, Chibuzor A Okoronkwo, Harrison Duru, Inelefo R Iyayi, Tinuade O Adeakin-Dada, Natalie O Doherty

**Affiliations:** 1 Family Medicine, Larkin Community Hospital Palm Springs Campus, Hialeah, USA; 2 Family Medicine, Medficient Health Systems, Laurel, USA; 3 Family Medicine, Lakeside Medical Center, Belle Glade, USA; 4 Family Medicine, University of Alberta Hospital, Edmonton, CAN; 5 Anaesthesia, Federal Medical Centre Birnin Kebbi, Birnin Kebbi, NGA; 6 Internal Medicine, University of Benin, Benin City, NGA; 7 Community and Family Medicine, Windsor University School of Medicine, Brighton Estate, KNA; 8 Medicine and Surgery, Igbinedion University, Benin City, NGA

**Keywords:** respiratory sounds/wheezing, treatment outcome, united states, canada, asthma management, asthma diagnosis

## Abstract

Globally, asthma remains the most widespread chronic respiratory condition in children, with a larger proportion of children being affected by the condition. Regardless of the higher prevalence rates, the outcomes of pediatric asthma have remained inadequate, even as there are numerous preventable deaths (approximately 300 children in the United States and 250 children in Canada, annually). The characteristic symptoms of pediatric asthma include wheezing, cough, and shortness of breath that are characteristically triggered by several potential stimuli. However, several diagnostic challenges exist and have resulted in either overdiagnosis or underdiagnosis, making pediatric asthma diagnosis and management problematic. Effective management of asthma in children entails a holistic approach that encompasses non-pharmacological and pharmacological management, alongside self-management and educational aspects. Working with pediatric asthma patients and their families/caregivers is vital to promoting and realizing better asthma diagnosis and management outcomes. Educational guidelines regarding the best ways for effective treatment, avoidance of triggers, modifiable risk factors, and the actions that should be taken during chronic asthma attacks through individualized action plans are vital. Thus, the objective of this systematic review is to provide an overview of the latest guidelines on pediatric asthma diagnosis and management. In this regard, this review presents several similarities in existing pediatric asthma diagnosis and management guidelines in the United States and Canada. For instance, most guidelines and studies reviewed have proposed the use of objective tests for confirmation of asthma diagnosis, particularly in symptomatic individuals. The peak flow variability measurement, bronchodilator reversibility testing, and spirometry have also been proposed by the guidelines and studies, even as the recommendations regarding the timing and hierarchy of the objective test substantially vary between the guidelines and studies. We hope that the present review will be helpful to physicians and healthcare service providers working within pediatric health contexts.

## Introduction and background

Asthma is a chronic inflammatory disease affecting the airways characterized by variable airway obstruction and bronchial hyperreactivity leading to intermittent episodes of breathlessness, wheezing, coughing, and chest tightness that might vary in intensity over time. Although asthma affects over a quarter billion individuals globally, it remains the most widespread chronic condition in children and has been attributed to more than 1,000 deaths per day, most of which are preventable [1,2]. Globally, asthma affects approximately 14% of children [3]. The 2018 report on pediatric asthma prevalence in the United States, particularly in children below 18 years of age, reported a prevalence of 7.5%, with the most affected pediatric population being those aged between 5 and 14 years (8.6%), Puerto Ricans (17%), non-Hispanic Blacks (14.3%), low-income households (10.2%), and Northeasterners (8.9%) [4,5]. In Canada, the national asthma prevalence stands at 10.8%, with approximately 3.8 million Canadians aged above one year suffering from the condition [6].

In recent times, there have been several reports from North America, particularly the United States and Canada, highlighting a higher rate of asthma misdiagnosis in children, including under and overdiagnosis [6,7]. Thus, poor asthma diagnosis and management have been linked to several adverse effects on children and their families. For instance, in addition to leading to preventable mortality, poor asthma management is prone to result in affected children missing school days, having additional educational requirements, and having lower educational attainment compared to other children [8]. Moreover, the families and caregivers of pediatric asthma patients are also prone to miss work days, in addition to experiencing fiscal challenges due to asthma misdiagnosis and poor management, even as certain pediatric patients are likely to experience severe asthma symptoms and fatal attacks [9].

In the United States and Canada, pediatric asthma outcomes have been observed to be poor with substantial related morbidity and increased rates of hospital admissions, and, more pertinently, several preventable deaths every year. Worryingly, Martin et al. have disclosed that, in nearly all pediatric asthma cases, several significant avoidable contributing factors existed, and that such deaths might have been prevented [6,8-13]. Several factors have made the diagnosis and management of pediatric asthma challenging. The objective of this systematic review is to provide an overview of the latest guidelines on pediatric asthma diagnosis and management.

## Review

Methodology

To effectively gather the pertinent literature on guidelines for pediatric asthma diagnosis and management, an extensive search on various virtual databases, such as SCOPUS, PubMed, Google Scholar, Embase, and Web of Science, was conducted. Only studies and reports published between 2015 and July 2024 in the English language were selected. Moreover, the reports and articles selected included guidelines and epidemiological research that included de-identified participant data alongside diverse multicenter surveys, as well as review articles published in reputable journals. A comparison of studies was further conducted to identify duplicate articles and reports from comparable publication years, while studies that had increasingly logical details were included. Furthermore, several MeSH keywords were employed for the literature search, including asthma diagnosis, asthma management, Canada, United States, treatment outcome, and respiratory sounds/wheezing. The literature search yielded 1,256 studies.

Inclusion and Exclusion Criteria

After the identified duplicates were removed, relevant literature was further subjected to selection in three definite stages. The first stage included the screening of the titles and abstracts of the selected articles. The subsequent stage involved the removal/exclusion of articles deemed irrelevant to this study. The last stage involved a comprehensive full-text assessment of the selected literature to ensure that the correct and pertinent articles were designated and included. The three phases of literature screening were mainly conducted by a team of three independent reviewers. Potential discrepancies were resolved through consultations and consensus.

Additionally, the inclusion criteria included original studies, such as prospective cohort studies, crossover design studies, and randomized controlled trials, that satisfied the set criteria as follows: articles focusing on pediatric asthma diagnosis and management in the United States and Canada; articles on pharmacological and non-pharmacological management of asthma in children; and articles published in the English language between 2015 and 2024. At this stage, editorials, opinion pieces, narrative reviews, and funded clinical trials were excluded. The first assessment of the abstracts of the articles led to the removal of 347 articles. Further, the vital data drawn from the qualified selected literature were extracted in the following manner: (a) the study’s general attributes, including the authors’ names, study year and publishing year, and the sampling methods used; (b) the attributes of the study population, such as gender, age, race, sample size, and follow-up; (c) the type of intervention used and the intervention duration, in addition to the interventions proposed; and (d) the main findings of the study.

**Figure 1:**
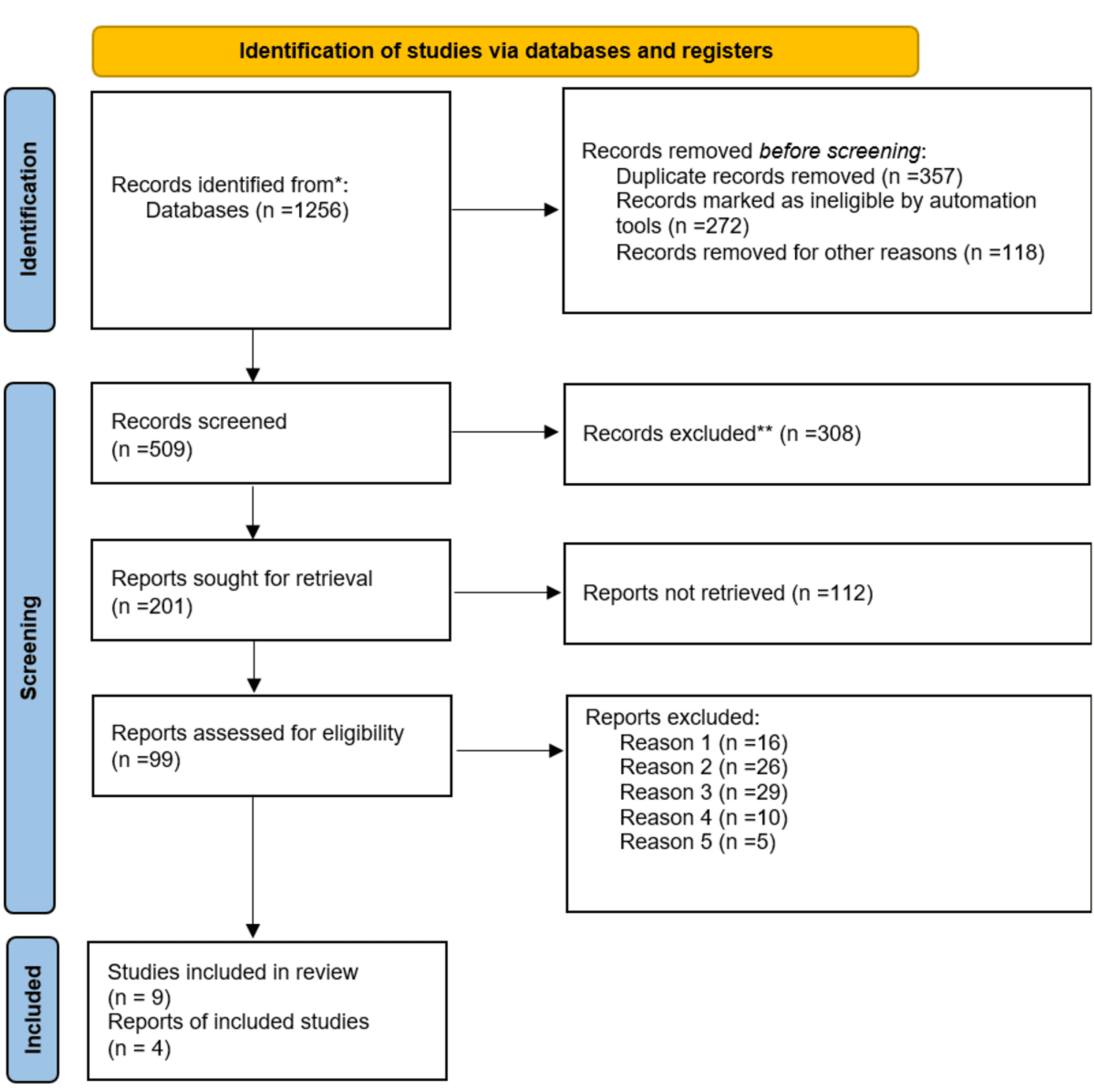
Preferred Reporting Items for Systematic Reviews and Meta-Analyses (PRISMA) flow diagram showing the study selection process.

Finally, this systematic review employed the Preferred Reporting Items for Systematic Reviews and Meta-Analyses (PRISMA) guidelines in the literature selection process. A total of 1,256 articles were retrieved after a comprehensive database search. The successive screening of the articles led to the elimination of 357 duplicate articles and 272 ineligible articles via automation, as well as an additional 118 articles excluded for other outstanding reasons, including non-alignment with this study’s objectives, and adult human-based studies. Additionally, non-peer-reviewed journal articles and dissertations were excluded alongside studies initially published in non-English languages. Consequently, 509 eligible articles underwent further screening leading to the exclusion of 308 articles. The researchers sought the remaining 201 articles for retrieval, with 112 articles being irretrievable. Therefore, 99 articles underwent assessment for appropriateness leading to the removal of an additional 86 articles after a full-text screening for various reasons, including irretrievable full-text even after contacting the authors (16 studies); preprints (26 studies); targeted interventions not assessed (29 studies); protocol (10 studies); and limitations not reported (5 studies). The PRISMA flow diagram presented in Figure 1 presents the article selection process. Table 1 presents a summary of the included articles.

**Table 1:** A summary of the studies included in this review.

Authors	Title of the study	Study objectives	Findings
Global Asthma Report [14]	The Global Asthma Report	To assess the prevalence, burden, and impact of asthma globally	Asthma affects nearly 339 million individuals globally, with considerable variations in both the prevalence and impact across varied nations and regions
Aaron et al. [15]	Underdiagnosis and overdiagnosis of asthma	To assess the prevalence alongside the impact of underdiagnosis and overdiagnosis of asthma	Asthma is often underdiagnosed, especially in children presenting with symptoms indicative of asthma who have not received any diagnosis. This often results in untreated asthma worsening over time
2022 GINA Report [16]	Global strategy for asthma management and prevention	To provide the most recent evidence and guidelines for the prevention, diagnosis, and management of asthma	The report offers novel treatment guidelines, including inhaled corticosteroid (ICS) use as the initial treatment for every patient with persistent asthma
United States Environmental Protection Agency (EPA) [17]	Expert Panel Report 3: guidelines for the diagnosis and management of asthma	To provide up-to-date and evidence-based recommendations regarding the diagnosis and management of asthma	The report has recommended refined diagnostic criteria for asthma, including spirometry and peak flow measurements use, as well as the assessment of asthma triggers and symptoms
Cloutier et al. [18]	2020 focused updates to the asthma management guidelines: a report from the National Asthma Education and Prevention Program Coordinating Committee Expert Panel Working Group	To provide focused updates regarding the 2007 National Asthma Education and Prevention Program (NAEPP) asthma management guidelines	The panel proposed ICS use based on an as-needed basis for specific patients with mild and persistent asthma as an option to everyday ICS use
www2.gov.bc.ca [19]	Asthma diagnosis, education, and management	To establish standardized criteria for precise diagnosis and management of asthma, as well as to ensure that healthcare workers can identify asthma in children early	Distinct guidelines and diagnostic tools were indicated to enhance the precision of asthma diagnosis in children, thereby minimizing misdiagnosis and underdiagnosis
Kwong and Bacharier [20]	Phenotypes of wheezing and asthma in preschool children	To comprehend the different patterns of wheezing and other asthma presentations in children and to assess how such phenotypes may predict long-term responses and treatment outcomes	Several distinct wheezing phenotypes were identified in preschool children, such as transient early wheezing, atopic wheezing/asthma, and non-atopic wheezing
The American Thoracic Society [21]	Asthma	To provide an inclusive resource on asthma diagnosis and management, covering the most recent guidelines, treatment approaches, and clinical studies	The guidelines highlighted the significance of adherence to treatment regimens and focused on precise conditions that included exercise-induced bronchoconstriction. Additionally, the guidelines emphasized customized treatment plans for enhancing asthma patient outcomes
Trottier et al. [22]	Managing an acute asthma exacerbation in children. Paediatr Child Health.	To deliver an up-to-date, evidence-based proposal for the management of severe asthma in children	Early recognition, management, and treatment of asthma exacerbations considerably enhance asthma outcomes. The researchers stressed the significance of early interventions that include apposite medications, such as inhaled corticosteroids and bronchodilators
Holguin et al. [23]	Management of severe asthma: a European Respiratory Society/American Thoracic Society guideline	To provide evidence-based proposals for the diagnosis and management of severe asthma in children and to tackle the intricacies and challenges of managing severe asthma	The study recommended biologic therapies, including anti-IgE, anti-IL-4/13, and anti-IL-5 agents, for children with definite phenotypes of chronic asthma, indicating substantial improvements in asthma symptom control and management
Murphy and Solis [24]	National Asthma Education and Prevention Program 2020 guidelines: What’s important for primary care	To provide focused and up-to-date guidelines on asthma management based on the most recent evidence, mainly in primary care contexts	The guidelines proposed ICS use on an as-needed basis for the effective management of mild and persistent asthma as an option to daily ICS use
Kuprys-Lipinska et al. [25]	New approach to intermittent and mild asthma therapy: evolution or revolution in the GINA guidelines?	To evaluate the GINA guideline updates, especially the recommendations for the management and treatment of patients with mild and intermittent asthma	The study highlighted the recommended use of the novel GINA guidelines, which stipulate the use of ICSs and long-acting beta-agonists for children with mild and intermittent asthma, even those who do not require everyday treatment
Johnson et al. [26]	Differential diagnosis of asthma. Allergy and asthma	To investigate the differential asthma diagnosis, concentrating on the differentiation of asthma from conditions with comparable symptoms such as wheezing, shortness of breath, and coughing	A substantial percentage of children diagnosed with asthma might be suffering from divergent conditions, resulting in incorrect treatment and poor care outcomes. Spirometry use, bronchodilator responsiveness testing, as well as imaging have been recommended as important tools in distinguishing asthma from conditions with similar symptoms

Risk of Bias and Quality of Included Studies

The articles selected were evaluated for quality and risk of bias using the Joanna Briggs Institute quality assessment checklist, with a mean quality score of 9. Thus, 10 articles were found to be of the highest quality with a score of ≥ 8.75, while the other three articles were found to be of moderate quality with scores ranging between 6 and 8.75. Therefore, none of the 13 articles identified was of poor quality. To reduce the risk of bias linked to individual studies, only publications with results from double-blind, randomized, and controlled trials were included. Duplicated titles identified from the in-depth search conducted on the different databases and with slight differences in the title entries and authors in the different databases were excluded.

Discussion

Asthma Diagnosis in Children

Asthma misdiagnosis in children has often been attributed to respiratory symptoms that are common within the age group. Such respiratory symptoms are always non-specific and represent viral respiratory tract infections [9,10], several of which might be prolonged with their clinical symptoms being comparable to asthma. Hence, an accurate asthma diagnosis in children is important given that overdiagnosis has frequently led to overtreatment using corticosteroids [11], with various implications concerning the cost of healthcare, the higher risk of unwarranted side effects, and, in certain instances, delays in the establishment of vital diagnostic alternatives [12]. Consequently, underdiagnosis along with undertreatment/management of pediatric asthma leads to unnecessary morbidity, increment in mortality, and poor quality of life, particularly in low-resource contexts [13].

Effective management of pediatric asthma requires confirmation of asthma diagnosis. Primary care physicians are normally consulted by several patients with numerous and diverse medical conditions every year. Moreover, physicians also experience challenges in quickly arriving at precise diagnoses within a limited time frame, and with restricted access to specialized investigations. Therefore, to ascertain accurate asthma diagnosis in children as early as possible, there is a need for physicians to sustain a higher index of suspicion in pediatric patients presenting with respiratory symptoms [14]. Asthma diagnosis is mainly based on the patient’s clinical history, physical examination compatible with asthma, and objective evidence indicating reversible airflow obstruction [6]. The widespread under and overdiagnosis of asthma has been attributed to the unavailability of objective lung function testing capable of demonstrating variable expiratory airflow limitation, which supports the diagnosis of asthma and aids in the exclusion of other potential causes [15]. The limited availability of pulmonary function tests (PFTs) in underserved and resource-poor communities in the United States and Canada, even as participation of children in spirometry is restricted by age. Thus, only children aged above five years can undergo PFTs for asthma diagnosis and follow-up management [15-17].

For care continuity, it is vital to ascertain that apt recording of the diagnosis occurs in the patient’s medical record, offering details regarding the diagnosis rationale, including the objective evaluation of airway inflammation and obstruction. Such details are normally absent in children’s medical records as well as in adult asthma patients [3]. Additionally, the patient’s medical records should include the details of the prescribed treatment, information offered to help the patient understand the severity of his/her condition, and written individual action plans that guide patients on how and when to seek assistance when needed, allowing healthcare professionals to adjust their treatment accordingly.

To aptly diagnose asthma in children aged 6-11 years, Global Initiative for Asthma (GINA) guidelines have recommended that a physician should confirm the asthma diagnosis before commencing the treatment, whenever feasible [16]. At present, no single test exists for confirming an asthma diagnosis [2]. As such, GINA guidelines recommend that the clinical diagnosis should commence with a history of respiratory symptoms, including wheezing, coughing, shortness of breath, and difficulties in breathing, which differ over time and in intensity [16]. Similar observations have been made by Environmental Protection Agency (EPA) guidelines, which maintain that asthma symptoms often worsen at night and during early morning, and are often triggered by several factors, including strong smells, laughter, exhaust fumes, cigarette fumes, exercise, viral infections, and exposure to allergens [17]. The other cardinal symptom of untreated asthma in children is the variable expiratory airflow [15]. When the history of a pediatric patient strongly indicates asthma, the diagnosis should be confirmed through the increment in forced expiratory volume in one second (FEV1), based on the spirometry measurement. For adults and adolescents, the confirmation of asthma diagnosis requires an increase of more than 200 mL and 12% from the baseline FEV1, which is recorded 15 minutes following a bronchodilator administration. Consequently, in children, the asthma diagnosis should be confirmed by an increase of over 12% from the baseline of the projected FEV1 value.

Moreover, as asthma is an increasingly variable disease, both reversibility and bronchodilator responsiveness might or might not exist during the initial testing of the lung function [9]. In instances where there is no documentation of spirometry during the first attempt, a repeat of the test should be conducted at another visit, especially when the patient is not only symptomatic but also when the bronchodilator medications have been withheld [18]. If not, an optional test should be performed. Normally, access to spirometry in primary care is always limited. However, the alternative technique entails instructing the patient or the patient’s caregiver to document the peak expiratory flow (PEF) every morning and late in the evening over two weeks in a diary or through the use of an electronic peak flow meter [18]. According to Gautier and Charpin, the measurement of PEF should be undertaken thrice on every occasion, with only the highest number being utilized [9]. Further, the variability of the diurnal PEF should be computed as every day’s biggest reading minus the lowest reading of the day and divided by the highest reading and lowest readings of the day’s mean. The result is then averaged over seven days. The excess diurnal PEF variability has been described as 13% of the average variability in children. The GINA guidelines further stipulate that in evaluating PEF, the physician should utilize the same meter for every reading, given that the variation between dissimilar PEF meters might be as high as 20% [16].

In children aged 6-11 years with undiagnosed asthma and who present usual expiratory airflow devoid of any considerable reversibility, the broncho-provocation test, which includes mannitol and methacholine, should be utilized in revealing the airway hyperresponsiveness, thereby supporting asthma diagnosis in such children. However, the bronchodilators must be withheld before conducting the challenge testing. Preferably, the variable expiratory airflow restriction should be demonstrated before initiation of the asthma controller treatment, except for clinically urgent circumstances, given the difficulty in confirming asthma diagnosis in children upon commencement of controller treatment [3]. Nonetheless, asthma diagnosis might additionally be confirmed in case of clinically significant FEV1 improvement by over 12% and over 200 mL, as well as a PEF improvement by over 20% after four weeks of treatment involving inhaled corticosteroid (ICS). Aaron et al. have recommended a different diagnostic criterion for asthma for children aged five years and below [15]. According to Chaplin, diagnosing asthma in children aged ≤5 years is a challenge, given that within this age group, recurrent wheezing has been acknowledged to be widespread and can be found in those without asthma, characteristically in children with infections of the upper respiratory tract [2]. Moreover, in this age group (≤5 years), regular airflow limitation assessment and bronchodilator responsiveness are not only difficult but also impracticable in primary care settings [2,15].

According to GINA guidelines, in children aged ≤5 years, asthma diagnosis should be based on aspects that include symptom patterns, the presence of risk factors, the exclusion of alternative diagnoses, and the therapeutic response to the controller treatment [16]. Thus, in children aged ≤5 years, asthma diagnosis is highly likely if one has a history of wheezing and coughing while exercising, crying and laughing, and devoid of respiratory tract infections; an allergic condition history, including food-related allergies, eczema, atopy, rhinitis allergy; and a history of asthma in first-degree relatives, along with clinical improvements after two to three months under controller treatment followed by worsening after treatment cessation [16,19]. Additionally, in preschool children with wheezing, the short-term symptom patterns have been used in phenotypes proposal, even though these have not proven to be clinically accurate and important in predicting asthma during later childhood [20]. Moreover, the Asthma Predictive Index (API) is a validated highly useful tool used in the screening and diagnosis of asthma in preschool children [16,19,20].

Asthma Severity Classification

The Expert Panel Report 3 (EPR-3) has broadly classified asthma severity into different categories based on the persistence and intermittence of the symptoms. Thus, it is recommended that children with intermittent asthma are treated based on the first step of the therapy, while patients with persistent and severe asthma be treated using the second to the sixth steps, reliant on whether or not their asthma condition is mild, moderate, or severe and persistent. Thus, the severity of asthma in children has been described as the inherent intensity of the condition, in addition to being mainly based on the lowest levels of therapy that enable control of the patient’s asthma. The control of asthma is mostly founded on aspects of impairment along with the future risk criterion for exacerbation. The ascertainment of the impairment is mainly done by the pediatric patient’s caregiver’s remembrance of the different symptoms, the functioning in the last two to four weeks, and spirometry findings. Consequently, the risk is determined through the frequency and number of exacerbations that require oral corticosteroid use. The severity of asthma is mainly allotted to the increasingly chronic category where many features and symptoms exist.

Asthma Management in Children

Numerous American and Canadian guidelines focusing on pediatric asthma have recommended that all asthma patients be treated using ICS-containing medications [16,19]. In this regard, GINA guidelines have recommended that children aged above five years and with asthma diagnoses should be managed using regular (in cases of mild asthma), as-required ICS-containing treatments to control the symptoms and prevent flare-ups and exacerbations/attacks [16]. Patients should also undergo reviews in three months following the initiation or alteration of the asthma treatment [2]. However, in children aged five years and below, ICS treatment is recommended only in instances where asthma diagnosis is increasingly probable even as the patient’s asthma symptoms are uncontrolled with approximately three or more episodes of wheezing in a year [21]. Additionally, an ICS trial is recommended in instances where asthma diagnosis is uncertain and the occurrence of the symptoms is more than every six to eight weeks. In this regard, Trottier et al. have asserted that the major asthma management components include the assessment of control and the risk of exacerbation/worsening; provision of self-management education for asthma, which includes a written action plan; identification of the triggers and discussion of environmental controls whenever applicable; and prescription of apt pharmacological therapy to aid in the realization and maintenance of control [22]. The other component of asthma management entails the assessment of the control and risk. In this regard, the Global Asthma Network maintained that, upon confirmation of a patient’s asthma or in instances where asthma is highly probable, assessment of the patient’s control of the asthma symptoms, assessment of the risk of exacerbation, and creation or modification of the patient’s treatment plan [14].

Further, several pediatric asthma guidelines have laid out asthma treatment and management regimens based on factors that include age, severity of the disease, and control requirements [23]. The National Asthma Education and Prevention Program guidelines have proposed the use of a stepwise approach to the pharmacologic therapy for pediatric asthma that commences with the most aggressive therapy required to realize control, followed by stepping down to the minimal therapy that aims to maintain control [17,24]. The objectives of such pharmacologic therapy include the minimization of nocturnal and daytime symptoms, the frequency of asthma episodes, as well as the employment of short-acting beta-agonists (SABAs), to enhance PEF to above 80% of individuals and permit pediatric patients to partake and maintain common activities devoid of producing adverse drug side effects [24].

Based on the review of the various guidelines, it can be noted that the proposals for pharmacological treatment and management of asthma are mostly based on the stepwise approach that utilizes shared decision-making in the realization and maintenance of asthma controls at a lower and effective therapeutic regimen [17]. Regarding the stepwise approach to asthma treatment, the National Asthma Education and Prevention Program (NAEPP) guidelines have provided several proposals for intermittent asthma (first step), persistent and mild asthma (second step), as well as modest to chronic and persistent asthma (third to fifth steps) [17]. Most of these recommendations relate to the novel recommended uses for as-required dual therapy with faster-acting bronchodilators alongside ICSs, and the administration of long-acting muscarinic antagonists together with adjunctive subcutaneous immunotherapy.

In children aged 0-4 years, the NAEPP guidelines stipulate that the most appropriate initial management approach entails the use of the PRN SABA [17]. Thus, in children with persistent wheezing, a treatment regimen administered over a 7-10-day period and comprising ICSs and PRN SABA should be initiated during the onset of the respiratory tract infection [14]. As a management strategy, the use of ICSs alongside PRN SABA aids in decreasing the exacerbation of asthma while also limiting systemic corticosteroid use. The second step involves the use of everyday low-dose ICSs administered as the key controller treatment alongside PRN SABA as the fast relief treatment. The recommended alternative regimen includes everyday cromolyn or montelukast and PRN SABA [19]. The thirdstep entails an everyday medium dosage of ICS as the controller treatment alongside PRN SABA for faster relief, even as the fourth step entails an everyday medium dose of ICSs and long-acting β2-agonist (LABA) as the controller treatment alongside PRN SABA for faster relief treatment. In this case, the alternative regimen includes an everyday medium dosage of ICS along with montelukast and PRN SABA [17]. The fifth step involves a higher daily dose of ICS-LABA as the controller treatment along with PRN SABA for fast relief, with the optional regimen being a higher everyday dosage of ICS with montelukast administered alongside PRN SABA for faster relief. At present, the guidelines have presented salbutamol/albuterol as the most preferred SABA medication while formoterol is the most preferred LABA medication [16,21]. Nevertheless, according to the American Thoracic Society (ATS) guidelines, the Food and Drug Administration has recently issued a warning about the use of montelukast owing to the medication’s adverse neuropsychiatric events [21]. It is recommended that pediatric patients (aged 0-4 years) should be reassessed after four weeks and step-ups made as necessary. The physician might consider stepping down the treatment in case the patient’s asthma is well-controlled over three consecutive months. The NAEPP guidelines for the management of asthma in children are shown in Table 2.

**Table 2:** National Asthma Education and Prevention Program guidelines for the management of asthma in children. ICS: inhaled corticosteroid; OCS: oral corticosteroid; SABA: short-acting beta-agonists; LABA: long-acting beta-agonists; LTRA: leukotriene receptor antagonist; anti-IL5: anti-interleukin-5 therapy

Controller options	Step 1	Step 2	Step 3	Step 4	Step 5
Preferred controller aimed at preventing exacerbations and controlling symptoms	Low-dose ICS taken when SABA is taken	Daily low-dose ICS (see table of ICS dose ranges for children)	Low-dose ICS-LABA, OR medium-dose ICS, OR very low-dose ICS-formoterol maintenance and reliever therapy (MART)	Medium-dose ICS-LABA, OR low-dose ICS-formoterol maintenance and reliever therapy (MART), OR add tiotropium or LTRA	Refer for phenotypic assessment ± higher dose ICS-LABA or add-on therapy
Other control alternatives/Options	Daily low-dose ICS	Daily LTRA, or low-dose ICS taken when SABA is taken	Low-dose ICS + LTRA	Add tiotropium or LTRA	Add-on anti-IL5, and add-on OCS, with consideration of the side-effects

Consequently, in children aged between 5 and 11 years, asthma management guidelines have recommended a stepwise approach that commences with the first step, in which the administration of PRN SABA is recommended [16,17,19] (Table 3). The second step entails the initiation of daily low-dose ICSs as the controller treatment that is supplemented by PRN SABA for faster relief, with the recommended alternatives including everyday leukotriene receptor antagonists (LTRA), PRN SABA, nedocromil, cromolyn, and theophylline. The third step involves the use of a daily low-dose ICS-formoterol combination for both control and faster relief, with the recommended alternatives including daily medium-dose ICS with PRN SABA, or low-dose ICS and LABA, LTRA, theophylline, and PRN SABA. Consequently, step 4 entails a daily medium dosage of ICS-formoterol for control and fast relief, and the alternative is a medium-dose ICS-LABA or ICS in combination with LTRA or theophylline and PRN SABA. The fifth step involves a daily high dosage of ICS-LABA and PRN SABA, with the alternatives being a high dose of ICS with LTRA or theophylline with PRN SABA. The sixth step involves a daily high dose of ICS-LABA along with oral systemic corticosteroids and PRN SABA, and LTRA or theophylline may be added.

**Table 3:** Stepwise approach for management of asthma in children aged 5-11 years. Each step: Patient education, environmental control, and management of comorbidities. Steps 2–4: Consider subcutaneous allergen immunotherapy for patients with allergic asthma.

Step 1	Step 2	Step 3	Step 4	Step 5	Step 6	Assess control
Preferred: Short-acting beta-agonist as needed	Preferred: Low-dose inhaled corticosteroid. Alternative: Cromolyn, leukotriene receptor antagonist, nedocromil, or theophylline	Preferred: Either Low-dose inhaled corticosteroid + either long-acting beta-agonist, leukotriene receptor antagonist, or theophylline or medium-dose inhaled corticosteroid	Preferred: Medium-dose inhaled corticosteroid plus long-acting beta-agonist or medium-dose inhaled corticosteroid plus either leukotriene receptor antagonist or theophylline	Preferred: High-dose inhaled corticosteroid plus long-acting beta-agonist. Alternative: High-dose inhaled corticosteroid plus either leukotriene receptor antagonist or theophylline	Preferred: High-dose inhaled corticosteroid plus long-acting beta-agonist plus oral systemic corticosteroid. Alternative: High-dose inhaled corticosteroid plus either leukotriene receptor antagonist or theophylline plus oral systemic corticosteroid	Step up if needed. First, check adherence, inhaler method, environmental control, and comorbid conditions. Step down if feasible and asthma is aptly controlled for no less than 3 months

For patients above 12 years, the guidelines recommend various stepwise approaches to asthma management [3,18,21]. The initial step entails the use of PRN SABA, while the second step entails the use of daily low-dose ICS along with PRN SABA, or PRN with SABA and ICS. The recommended alternatives include a daily dose of LTRA with PRN SABA, or nedocromil, cromolyn, zileuton, or even theophylline and PRN SABA, despite these medications being less preferred in the United States due to their potential adverse reactions. Consequently, the third step recommends daily low-dose PRN with ICS-formoterol. The proposed alternatives include a daily medium dose of ICS and PRN SABA, or a daily low dose of ICS and LABA, tiotropium, LTRA, and PRN SABA [3]. The fourth step recommends a daily PRN with a medium dose of ICS-formoterol, with alternatives being medium-dose ICS-LABA or ICS and LAMA and PRN SABA. The fifth step involves the administration of a medium to high dose of ICS-LABA together with LAMA and PRN SABA, or the administration of an everyday high dosage of ICS with LTRA and PRN SABA. Lastly, the sixth step recommends the administration of a daily high dose of ICS-LABA together with oral systemic corticosteroids and PRN SABA [3,18].

The other notable asthma management approach recommended for children is the Single Maintenance and Reliever Therapy (SMART). As an approach, SMART entails the use of ICSs and LABA for everyday and emergency treatment and is recommended as the most appropriate therapy for patients aged four years and below with poorly controlled asthma and on medium or low doses of daily ICSs only [25]. As the most preferred LABA, formoterol has a faster action onset and may be administered twice daily. SMART aids in reducing symptom exacerbation and the overall use of corticosteroids. Patients on SMART have a reduced risk of growth suppression compared to those on higher everyday doses of ICS treatment [25]. As a result, pediatric patients with poorly controlled asthma on everyday ICS and LABA maintenance therapy are recommended for consideration for placement on SMART before stepping up the treatment [25].

Another notable aspect of asthma management and treatment in children that has been recommended by various guidelines is the clinical utility of fractional exhaled nitric oxide (FeNO) testing [17,19,21]. Thus, the approach involves the calculation of nitric oxide within the exhaled breath and the approximation of the airway inflammation level. In children with asthma, FeNO is considered an important indicator of type 2 airway eosinophilic or bronchial inflammation. Being non-invasive and quantitative, FeNO is considered and recommended as a safe and straightforward method for airway inflammation estimation. For pediatric patients aged five years and above who have been diagnosed with asthma following extensive assessment, FeNO measurements have been observed to assist as an adjunct to both diagnosis and response to treatment [3]. Moreover, FeNO testing is particularly important in children with asthma and whose spirometry testing cannot be accurately tested. In children aged between 5 and 12 years, FeNO levels of >35 ppb are indicative of T2 inflammation, and such patients are increasingly prone to respond better to corticosteroid treatment [3]. FeNO has equally been endorsed by ATS clinical practice guidelines as an adjunct for both the diagnosis and determination of the likelihood of corticosteroid responsiveness [21].

Cloutier et al. reported that despite the recommendation of systemic corticosteroid use in certain circumstances, given their effectiveness in resolving severe asthma symptoms and exacerbations, the available recent evidence has offered a cautionary note [18]. Even as the adverse outcomes of long-term usage of systemic corticosteroids have been widely acknowledged, a growing body of evidence has indicated that even recurrent, brief dosing durations, for instance, three to seven days, in persons with asthma, have been linked to an array of adverse health outcomes [12,24]. The adverse effects include substantial increments in the risk of osteoporosis and osteoporotic fractures, pneumonia, sleep apnea, heart failure, cataracts, myocardial infarctions, hypertension, type 2 diabetes, and other disorders, in addition to increased healthcare costs [24].

Bronchial thermoplasty is another pediatric asthma management approach that has been widely used in the management of asthma in children [17,19]. As a physical modality, bronchial thermoplasty is mainly used as an aspect of bronchoscopy, which utilizes radio waves to reduce the airway smooth muscle mass. Nevertheless, the NAEPP 2020 focused updates proposed against bronchial thermoplasty use in children and adults with persistent asthma, as well as those with FEV1 levels between <50% and 60% or fatal asthma. Thus, bronchial thermoplasty is mainly recommended for patients whose asthma is poorly controlled and whose caregivers place a higher value on possible advantages and a lower value on potential harm. The probable benefits include improvements in the health-related quality of life along with a considerable reduction in asthma exacerbations, while the potential harms include the short-term worsening of symptoms and indefinite long-term adverse effects.

Finally, education and compliance are also among the most important aspects of asthma management in children, as recommended by all guidelines [16,17,19]. Thus, there is a need for education for both patients and caregivers to focus on not only the identification but also the avoidance of asthma triggers, comprehension of the use of various prescribed medications, the significance of monitoring and compliance, and the apt use of various inhalation devices [26]. Everyday self-management plans that offer the necessary guidance for patients during peak flow monitoring, symptom reporting, and medication use have been recommended by the guidelines reviewed [16,19,24]. Additionally, emergency action plans have also been acknowledged as helpful in the identification of exacerbations and the delineation of the necessary actions that should be taken during asthma attacks. It has been recommended that such emergency plans should be developed by physicians in consultation with the child’s caregivers, and the patients in case of older patients, and offered to them in a written form [19]. Regarding compliance, it is noteworthy that poor compliance is a key challenge in the management of pediatric asthma, which can be attributed to several factors, including the medication administration route (more preference is given to inhaled medications as opposed to oral medications), dosing frequency (once daily regimens are more preferred), the risk of side effects, and medication effects (slower action onset and long duration during discontinuance are poorly adhered to) [7]. Most pediatric patients with asthma are incapable of mastering the proper use of a metered-dose inhaler (MDI) even following repeated training, and upon succeeding in mastering the apt MDI techniques, approximately only 15% of the administered medications can reach the lungs [7]. Therefore, spacers have been recommended as they make the use of MDIs easier and have become essential asthma management tools in children below six years of age [7]. To attain the desired effectiveness levels, nebulizers and MDIs with facemasks have been recommended for children aged five years and below, especially during asthma emergencies. Further, dry powder inhalers have been recommended for use by children who are capable of demonstrating sufficient inhalation velocity through the use of the training whistle [18].

## Conclusions

Several extant pediatric guidelines have proposed the usage of objective tests in confirming asthma diagnosis, especially in symptomatic individuals. The measurements of peak flow variability, bronchodilator reversibility testing, and spirometry have additionally been recommended by the guidelines. Nevertheless, this review has indicated that the recommendations on the timing and hierarchy of the objective test substantially vary between the guidelines. The results of this include the variation of diagnostic tests employed across divergent healthcare contexts in Canada and the United States. Importantly, it is noteworthy that, at present, no child-focused, evidence-based asthma diagnosis guideline exists, with the normal approach involving the production of joint pediatric and adult asthma diagnosis and management guidelines. This often results in adult data extrapolation, especially in instances where there is no evidence in children. Moreover, the tests utilized in adult asthma patients under investigation might not prove to be effective and appropriate in pediatric patients, even as the best cut-offs for most such tests might not be similar in adults and children. This, therefore, makes the need for and development of child-focused evidence-based pediatric asthma diagnosis and management guidelines essential.
